# Risk Factors for the Presence of Chikungunya and Dengue Vectors (*Aedes aegypti* and *Aedes albopictus*), Their Altitudinal Distribution and Climatic Determinants of Their Abundance in Central Nepal

**DOI:** 10.1371/journal.pntd.0003545

**Published:** 2015-03-16

**Authors:** Meghnath Dhimal, Ishan Gautam, Hari Datt Joshi, Robert B. O’Hara, Bodo Ahrens, Ulrich Kuch

**Affiliations:** 1 Nepal Health Research Council (NHRC), Ministry of Health and Population Complex, Kathmandu, Nepal; 2 Biodiversity and Climate Research Centre (BiK-F), Senckenberg Gesellschaft für Naturforschung, Frankfurt am Main, Germany; 3 Institute for Atmospheric and Environmental Sciences (IAU), Goethe University, Frankfurt am Main, Germany; 4 Institute of Occupational Medicine, Social Medicine and Environmental Medicine, Goethe University, Frankfurt am Main, Germany; 5 Natural History Museum, Tribhuvan University, Swayambhu, Kathmandu, Nepal; United States Army Medical Research Institute of Infectious Diseases, UNITED STATES

## Abstract

**Background:**

The presence of the recently introduced primary dengue virus vector mosquito *Aedes aegypti* in Nepal, in association with the likely indigenous secondary vector *Aedes albopictus*, raises public health concerns. Chikungunya fever cases have also been reported in Nepal, and the virus causing this disease is also transmitted by these mosquito species. Here we report the results of a study on the risk factors for the presence of chikungunya and dengue virus vectors, their elevational ceiling of distribution, and climatic determinants of their abundance in central Nepal.

**Methodology/Principal Findings:**

We collected immature stages of mosquitoes during six monthly cross-sectional surveys covering six administrative districts along an altitudinal transect in central Nepal that extended from Birgunj (80 m above sea level [asl]) to Dhunche (highest altitude sampled: 2,100 m asl). The dengue vectors *Ae*. *aegypti* and *Ae*. *albopictus* were commonly found up to 1,350 m asl in Kathmandu valley and were present but rarely found from 1,750 to 2,100 m asl in Dhunche. The lymphatic filariasis vector *Culex quinquefasciatus* was commonly found throughout the study transect. Physiographic region, month of collection, collection station and container type were significant predictors of the occurrence and co-occurrence of *Ae*. *aegypti* and *Ae*. *albopictus*. The climatic variables rainfall, temperature, and relative humidity were significant predictors of chikungunya and dengue virus vectors abundance.

**Conclusions/Significance:**

We conclude that chikungunya and dengue virus vectors have already established their populations up to the High Mountain region of Nepal and that this may be attributed to the environmental and climate change that has been observed over the decades in Nepal. The rapid expansion of the distribution of these important disease vectors in the High Mountain region, previously considered to be non-endemic for dengue and chikungunya fever, calls for urgent actions to protect the health of local people and tourists travelling in the central Himalayas.

## Author Summary

The local transmission of dengue fever was confirmed in five lowland urban areas in 2006, along with the first report of the primary vectors of dengue virus, *Aedes aegypti* mosquitoes. Subsequent studies revealed a wide distribution of *Ae*. *aegypti* in 2009, and the first locally acquired dengue fever case in Kathmandu, the capital city of Nepal, during an epidemic in 2010. These records of a rapid expansion of dengue viruses and their primary vector, *Ae*. *aegypti*, in the Middle Mountain region and the more pronounced warming of mountains prompted us to investigate the altitudinal distribution and determinants of the abundance of dengue virus vectors in central Nepal. The first local transmission of chikungunya virus was recently reported from central Nepal in 2013. In this study, we document the distribution of *Ae*. *aegypti* and the secondary vector of dengue viruses, *Aedes albopictus*, from the lowlands (80 m) up to 2,100 m altitude in Dhunche, Rasuwa district. The climatic variables rainfall, temperature and relative humidity were significant predictors of their abundances. The distribution extension of these important disease vectors in the High Mountain region calls for urgent actions to protect the health of local people and tourists travelling in the central Himalayas.

## Introduction

*Aedes* (*Stegomyia*) *aegypti* (L.) and *Aedes* (*Stegomyia*) *albopictus* (Skuse) are known primary and secondary vectors of dengue virus (DENV) and several other arboviruses [1–4]. These species are also the known principal vectors of chikungunya virus (CHIKV) transmission [4–6]. *Aedes albopictus* has been recorded for Nepal since the 1950s [7,8], but the introduction of *Ae*. *aegypti* and its involvement in dengue fever (DF) outbreaks in Nepal are very recent events with *Ae*. *aegypti* first detected and dengue first outbreak reported in 2006 [9–12]. The presence of immature stages of *Ae*. *aegypti* and *Ae*. *albopictus* has already been reported from different areas of the Middle Mountain region, including Kathmandu Valley, average altitude 1,350 m above sea level[asl] [9,10,13,14]. Autochthonous cases of DF along with the circulation of all four DENV serotypes were first reported in 2006 during an outbreak that mostly affected urban areas of the Terai lowlands [12], and an autochthonous case from Nepal’s capital city Kathmandu was first reported during an epidemic in 2010 [15]. Although chikungunya fever (CHIK) cases have not previously been reported in Nepal, the first local transmission of CHIKV was recently reported in the Middle Mountain region of central Nepal in 2013 [16]. Moreover, a simultaneous circulation of DENV and CHIKV has been reported in many recent studies [6,17]. These findings along with reports of DF cases from different districts of the Middle Mountain region, with the majority of cases from central Nepal [9,14], suggest that CHIKV and DENV have already expanded their distribution into higher altitudes of Nepal. Despite frequent outbreaks of DF, no previous record of CHIK cases can be attributed to a lack of differential diagnosis in Nepal [16]. The expansion of CHIKV and DENV vectors into higher altitudes may accelerate because the observed warming is more pronounced in the mountain regions of Nepal compared to the lowlands of the Terai and Siwalik hill regions [18–20].

Over decades, the environment of Nepal has changed, largely due to urbanization, large-scale population movements and land conversion into newly inhabited areas. The favorable habitats that are available to vector mosquitoes differ with climatic factors, elevation and vegetation. Sampling approaches that include these factors in locations within the complex environment may provide valuable information about the altitudinal distribution of CHIK, DF and other vector-borne diseases in different physiographic regions [21] and a variety of productive larval habitat types. In spite of its prime importance, there is no information on the altitudinal distribution and other risk factors for the presence of these diseases in Nepal. In fact, the distribution of CHIKV and DENV vectors in different geographic areas of Nepal, with particular reference to their altitudinal or environmental distribution has not been studied.

In the absence of licensed vaccine [22,23] and specific treatment of infection with either virus [4,23,24], efforts should be made to reduce human-mosquito contact or eliminate vector populations. In this regard, control measures should be focused on eliminating the immature stages of the mosquitoes and their larval developmental sites [24]. Therefore, the present study used an altitudinal transect of central Nepal to update the information on altitudinal distribution, explore risk factors for the presence of CHIKV and DENV, and assess the effects of climatic variables on the abundance of the vectors.

## Materials and Methods

### Study sites

**Fig 1:**
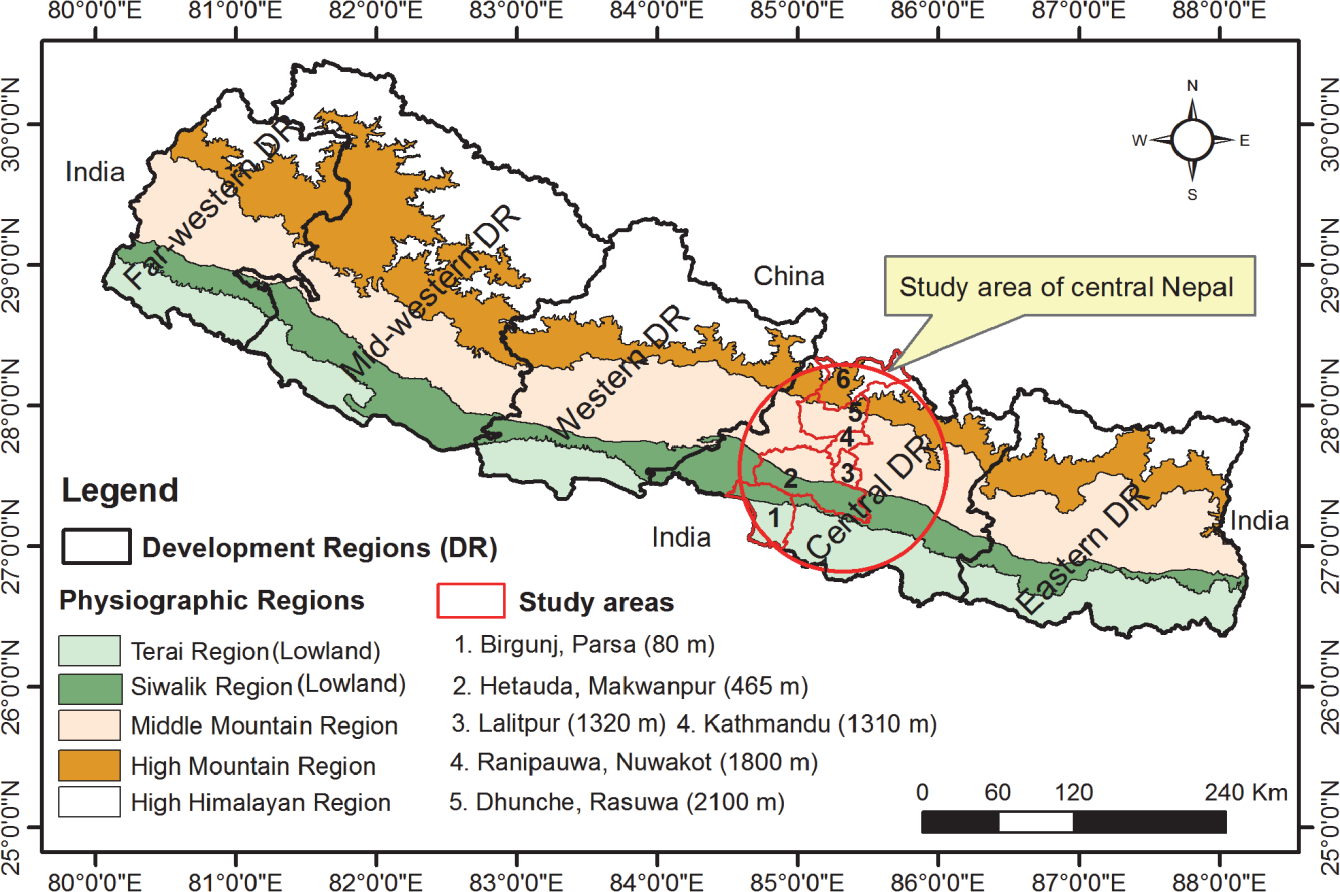
Map of study area. The map shows physiographic regions, development regions and study districts (sites) along an altitudinal transect from the lowlands (Birgunj; 80 m above sea level) to the High Mountain region (Dhunche; 2,100 m above sea level) in central Nepal. This map is updated from Dhimal et.al [25].

The study included an altitudinal transect of six administrative districts of the Central Development Region (hereafter referred to as central Nepal). Out of the total of 19 administrative districts we selected two districts –Nuwakot (Ranipauwa) and Rasuwa (Dhunche)– from the High Mountain region, two districts –Kathmandu and Lalitpur– from the Middle Mountain region, one district –Makwanpur (Hetauda)– from the Siwalik region, and one district –Parsa (Birgunj)– from the Terai region. These administrative districts have major cities/towns as well as trade routes extending from the Indian border in the south to the Chinese border in the north as shown in the map (Fig. 1). Except for Lalitpur, a detailed description of the study sites is given in Dhimal et al.[25] and socio-economic characteristics of the study sites in Dhimal et al. [26]. Lalitpur is a sub-metropolitan city adjoining the Kathmandu metropolitan city. It has 227,000 inhabitants residing in 55,000 households and a population density of 15 people per km^2^ [27]. The access to piped water supply and the climatic conditions of Lalitpur are similar to that of Kathmandu.

### Sampling and entomological surveys

In the first phase, six districts from central Nepal were selected along an altitudinal transect. In the second phase, it had been planned that at least 30 houses/premises would be sampled randomly each month from each study site. Hence, the targeted sample size was 30 houses x 6 districts x 6months = 1080. However, based on the availability of wet containers in different months, the number of premises inspected differed in each visit (due to fluctuation in number of water holding containers) and site resulting in a total of 834 houses/premises and 2,045 wet containers surveyed. Surveys for immature stages of mosquitoes were conducted in all of the study communities ranging from 80 m to 2,100 m asl in central Nepal. A door-to-door entomological survey was performed by two entomological teams, each consisting of three personnel, from September 2011 to February 2012. The area within the urban agglomerations or rural hamlets was selected and searched for all potential larval habitats, i.e., wet containers that held water for more than one day and seemed to be able to maintain this condition for more than 48 h. In each locality, the survey spots were in different directions. However, because of safety reasons and practical difficulties, the sampling frame excluded overhead tanks set up for water storage purposes, drainages and septic tanks. In order to minimize the potential confounding effects of temporal variation, parallel surveys were conducted in lower altitudes and higher altitudes (i.e., High and Middle Mountains by one team, and Terai lowlands and Siwalik hills by another team in each month).

Each selected house was geo-referenced using a portable global positioning system (GPS) device (Garmin eTrexH) and all accessible artificial larval developmental sites such as discarded tires, metal drums, plastic drums, other metal containers, plastic buckets, flower pots, mud pots, cement tanks, tree holes, rocks and other plastic containers in indoor and outdoor locations were inspected using dippers, as described elsewhere [10,13,28,29]. The equipment used for collecting *Aedes* spp. larvae consisted of a standard mosquito (400 ml capacity) dipper with extendable handle, pipette (10 ml), spoon and torchlight. All of the potential larval developmental sites were examined using a flashlight. If the flower pot was too small for the dipper to be inserted, the water contained in the flower pot was poured into the dipper instead. Indoor or intra-domiciliary refers to areas within the house or building under its roof whereas outdoor or peri-domiciliary refers to the exterior of the building, albeit limited to the immediate vicinity within 10 m from the house. All trash near and around dwellings (within 10 m from the selected house) was also examined. All live immature mosquitoes were collected using dippers and placed into vials/plastic cups covered with thin white pieces of muslin clothes labeled according to the container type, locality and date of collection. All live mosquito larvae collected were transported to the Natural History Museum, Tribhuvan University, Swayambhu, Kathmandu, where they were reared until adult emergence.

Adult mosquitoes emerged from the reared larvae and pupae were identified using taxonomic keys and by reference to morphological descriptions [7,30,31], as males and females belonging to the following taxonomic groups: (1) *Ae*. *aegypti*, (2) *Ae*. *albopictus*, (3) *Culex quinquefasciatus*, (4) *Aedes* sp. (exclusive of *Ae*. *aegypti*, *Ae*. *albopictus*), (5) others and (6) unidentified damaged specimens. Entomological indices were calculated as per sample size and techniques described in the World Health Organization Southeast Asia Regional Office (WHO SEARO) guidelines [24].

A container was recorded as positive for *Ae*. *aegypti* or *Ae*. *albopictus* when at least one immature of either species was observed. Likewise, a house or premise was recorded as positive when at least one larval developmental container contained immature stages of *Ae*. *aegypti* or *Ae*. *albopictus*.

### Weather data

Daily rainfall, temperature and relative humidity data at nearby meteorological stations from the vector survey areas (1–7 km) were obtained from Department of Hydrology and Meteorology (DHM), Government of Nepal, to compute the climatic variables that had prevailed one month preceding collections. Using the data on these climatic variables, we assessed their association with the abundance of each species of mosquito.

### Statistical analysis

The data were entered into Microsoft Excel spreadsheets and analysed using R computing software [32]. Our collected count data sets were over dispersed indicating the errors were not normally distributed. Therefore, we used generalized linear models (GLMs) to investigate the association between climatic variables and the abundance of mosquitoes of each species. We defined abundance as the number of individuals of a species per container and used monthly mean temperature, monthly total rainfall and monthly relative humidity as predictor variables. For each species we fitted GLMs assuming a negative binomial distribution and log link function, using the “MASS” package in R [33]. The GLM using a negative binomial distribution is reported to be a robust analysis especially with respect to over dispersed count data sets [34–36].

We modelled the presence or absence of larvae of *Ae*. *aegypti*, or *Ae*. *albopictus*, or both species co-occurrence as binary dependent variables using the “epicalc” package in R [37]. The predictor variables we used were physiographic region, month of collection, collection station, container type and their possible two way interactions. All variables that were significantly associated at family error rate *P*<0.05 were introduced into multiple logistic regressions and the best model was selected using Akaike’s information criterion (AIC). There was no evidence that co-linearity between the explanatory variables was a problem [all variance inflation factors (VIFs) <2]. A variogram of the model residuals did not show evidence for spatial autocorrelation [38]. We also did not find any evidence of significant temporal autocorrelation in model residuals.

We also aimed to document the presence of other mosquitos, e.g., *Cx*. *quinquefasciatus*, an important vector of lymphatic filariasis (LF), if captured during the collection of *Aedes* mosquitoes. However, we did not perform detailed statistical analyses of LF vector because some important potential habitats of *Cx*. *quinquefasciatus* such as drainage, toilets and polluted water containers were not inspected due to their excessive abundance in some areas especially of the lowlands.

*Stegomiya* indices: House (or Premises) Index (HI), the percentage of houses infested with larvae and /or pupae; Container Index (CI), the percentage of positive containers per 100 examined wet containers and Breteau Index (BI), the number of positive containers per 100 houses and their 95% confidence interval (CI) for mosquitoes of each species were calculated using an exact binomial test.

### Ethics statement

Ethical approval for conducting this study was granted by the Ethical Review Board (ERB) of the Nepal Health Research Council (NHRC).

## Results

### *Stegomyia* indices, species composition and altitudinal distribution of chikungunya and dengue vectors

From September 2011 to February 2012, a total of 2,045 water holding containers belonging to 834 households or premises were investigated for mosquito larvae. Altogether 3,241 larvae were recorded in this survey. Most of the mosquitoes (39.5%) were *Ae*. *aegypti*, and the second most abundant species was *Cx*. *quinquefasciatus* (16.1%) followed by *Ae*. *albopictus* (4.6%), other *Aedes* sp. (4.6%), and other mosquito species (9.9%). Among them, less than 1% were pupae. We could not identify 288 (8.6%) damaged immature specimens.

All three common species, *Ae*. *aegypti*, *Ae*. *albopictus*, and *Cx*. *quinquefasciatus*, were collected at elevations ranging from lowland Terai (∼85 m asl) to 2,100 m asl in Dhunche, High Mountain region. *Aedes aegypti* and *Ae*. *albopictus* were commonly found up to 1,350 m in Kathmandu valley, and present but rarely found from 1,750 m to 2,010 m in Ranipauwa and Dhunche, whereas *Cx*. *quinquefasciatus* was commonly found throughout our study transect (highest altitude studied 2,100 m als). The *Stegomiya* indices of *Ae*. *aegypti* and *Ae*. *albopictus* by physiographic region are given in Table 1. The *Stegomyia* indices of *Ae*. *aegypti* decreased with increasing elevation. There was also an inverse relationship between the presence of *Ae*. *albopictus* and altitude except for the very low altitude site (< 100m). The highest BI of *Ae*. *albopictus* was recorded in the Siwalik region followed by the Middle Mountain region. We have summarized data of *Cx*. *quinquefasciatus* in the Supplementary Table (S1 Table).

**Table 1:** *Stegomyia* indices of *Aedes aegypti* and *Aedes albopictus* in central Nepal.

Localities	Terai (< 90m)	Siwalik (400–500m)	Middle Mountain (1300–1400m)	High Mountain (1700–2100m)	Total
Houses/premises inspected	176	183	186	289	834
Wet containers inspected	493	488	560	504	2045
***Ae*. *aegypti*** number	672	421	176	12	1281
HI (95% CI)	47.7 (40.2–55.4)	46.4 (39.1–53.9)	22.0 (16.4–28.8)	1.4 (0.44–3.7)	
CI (95% CI)	25.2 (21.5–29.2)	24.8 (21.1–28.9)	11.1 (8.7–14.0)	1.8 (0.3–2.2)	
BI (95% CI)	70.5 (63.0–76.9)	66.1 (58.7–72.8)	33.2 (26.7–40.66)	1.4 (0.44–3.7)	
***Ae*. *albopictus*** number	59	270	176	14	522
HI (95% CI)	7.4 (4.2–12.6)	26.8 (20.6–33.9)	21.5 (16.0–28.2)	2.4 (1.1–5.1)	
CI (95% CI)	5.5 (3.7–8.0)	12.3 (9.6–15.6)	9.1 (6.9–11.9)	1.4 (0.6–3.0)	
BI (95% CI)	15.3 (10.5–21.7)	32.8 (26.1–40.1)	27.4 (21.3–34.5)	2.4 (1.1–5.1)	

HI: House Index

CI: Container Index

BI: Breteau Index

### Risk factors for the presence of chikungunya and dengue vectors

About 15.2% of the wet containers contained *Ae*. *aegypti*, 7.1% *Ae*. *albopictus*, and 2.9% both *Ae*. *aegypti* and *Ae*. *albopictus*. Container type was significantly associated with the presence of *Ae*. *aegypti* (*P*<0.001), *Ae*. *albopictus* (*P*<0.01) and the co-occurrence of both *Ae*. *aegypti* and *Ae*. *albopictus* (*P*< 0.05) (Table 2). However, the addition of container type to the multivariate model did not increase the fit of the models for both species and their co-occurrence, so this predictor was subsequently dropped in all models.

**Table 2:** Container types associated with the presence of immature stages of *Aedes aegypti*, *Aedes albopictus* and their co-occurrence in central Nepal.

Category	Examined	*Ae*. *aegypti* (*P* < 0.001)		*Ae*. *albopictus* (*P* = 0.001)		Co-occurrence (*P* = 0.011)	
Container types	wet containers	Positive (%)	OR (95% CI)	Positive (%)	OR (95% CI)	Positive (%)	OR (95% CI)
Plastic drum	199	5.0	1.00	2.5	1.00	0.5	1.00
Cemented tank	15	6.7	0.94 (0.11–8.02)	0.0	ND	0.0	ND
Discarded tire	1116	18.7	7.42 (3.64–15.12)	7.9	4.59 (1.8–11.74)	3.3	14.73 (2–108.51)
Flower pot/base	62	9.7	2.94 (0.96–8.95)	4.8	2.46 (0.56–10.87)	1.6	3.97 (0.24–65.19)
Metal drum	376	12.0	2.26 (1.06–4.81)	8.2	2.8 (1.06–7.43)	3.7	6.84 (0.89–52.58)
Mud pot	8	36.4	8.81 (2.1–37.03)	54.5	16.4 (3.48–77.21)	18.2	31.75 (2.59–388.55)
Plastic bottle	236	14.4	2.42 (1.07–5.48)	4.2	1.47 (0.47–4.61)	2.1	4.64 (0.53–40.21)
Plastic pot	8	12.5	2.2 (0.24–20.35)	25	9.84 (1.52–63.7)	0	ND
Unused bucket	11	9.1	1.32 (0.15–11.52)	0	ND	0	ND
Miscellaneous	11	0	ND	0	ND	0	ND
Total	2045	15.2		7.1		2.9	

ND: not determined

OR: Odds ratio

Miscellaneous includes tree hole, discarded aluminum utensils, steel pot, polythene bag, plastic mug, natural pond, metal plate, metal can, iron kettle.

Table 3 shows significant risk factors of the presence of *Ae*. *aegypti* in multivariate logistic regression analysis. After adjusting for confounding factors in multiple regression, the log odds of the presence of *Ae*. *aegypti* reduced with altitude, from lowland Terai to the High Mountain region. It was also most common between October and December. Finally, the odds of finding *Ae*. *aegypti* immatures outdoors was 3.53 times higher than indoors (aOR = 2.49, 95% CI = 2.41–5.16, *P*<0.001).

**Table 3:** Multivariate logistic regression analysis for the presence of *Aedes aegypti* larvae and pupae.

Predictor variables	Wet containers	Positive (%)	OR (95%CI)	aOR (95%CI)	*P*
**Physiographic region**					
Terai	493	25.1	1.00	1.00	
Siwalik	488	24.8	0.89 (0.6–1.31)	1.08 (0.71–1.66)	0.714
Middle Mountain	560	11.1	0.30 (0.19–0.47)	0.28 (0.17–0.45)	< 0.001
High Mountain	504	0.8	0.02 (0.01–0.05)	0.01 (0–0.04)	< 0.001
**Month**					
Sep-11	554	13	1.00	1.00	
Oct-11	357	21	1.79 (1.09–2.96)	2.36 (1.27–4.38)	0.006
Nov-11	290	24.1	2.20 (1.34–3.6)	1.37 (0.78–2.41)	0.269
Dec-11	186	27.4	2.49 (1.47–4.2)	1.26 (0.69–2.3)	0.447
Jan-12	306	7.5	0.89 (0.5–1.57)	0.45 (0.24–0.86)	0.016
Feb-12	352	5.7	0.64 (0.35–1.15)	0.46 (0.23–0.89)	0.021
**Station**					
Indoor	763	10	1.00	1.00	
Outdoor	1282	18.33	2.49 (1.8–3.45)	3.53 (2.41–5.16)	< 0.001

OR: Odds ratio

aOR: Adjusted odds ratio

Table 4 presents the significant risk factors of the presence of *Ae*. *albopictus* in multivariate logistic regression analysis. *Aedes albopictus* was most prevalent in regions at intermediate altitudes (i.e., Siwalik hills and Middle Mountain region), and was least prevalent in January and February. As with *Ae*. *aegypti*, *Ae*. *albopictus* was more likely to be found outdoors (aOR = 2.03, 95% CI = 1.3–3.18, *P*<0.01).

**Table 4:** Multivariate logistic regression analysis for the presence of *Aedes albopictus* larvae and pupae.

Predictor variables	Wet containers	Positive (%)	OR (95%CI)	aOR (95%CI)	*P*
**Physiographic region**					
Terai	493	3.2	1.00	1.00	
Siwalik	488	13.3	4.16 (2.18–7.93)	5.19 (2.66–10.1)	< 0.001
Middle Mountain	560	9.1	2.89 (1.49–6.61)	3.17 (1.61–6.24)	< 0.001
High Mountain	504	1.4	0.31 (0.12–0.8)	0.30 (0.12–0.77)	0.013
**Month**					
Sep-11	554	6.49	1.00	1.00	
Oct-11	357	11.2	1.30 (0.7–2.41)	1.35 (0.69–2.62)	0.38
Nov-11	290	11.4	1.53 (0.84–2.8)	1.05 (0.55–1.99)	0.888
Dec-11	186	10.8	1.43 (0.74–2.77)	0.78 (0.39–1.58)	0.496
Jan-12	306	2.6	0.45 (0.2–1.03)	0.28 (0.12–0.66)	0.004
Feb-12	352	2.3	0.39	0.3 (0.13–0.72)	0.007
**Station**					
Indoor	763	6.7	1.00	1.00	
Outdoor	1282	7.3	1.51(1.2–2.28)	2.03 (1.3–3.18)	0.002

OR: Odds ratio

aOR: Adjusted odds ratio

**Fig 2:**
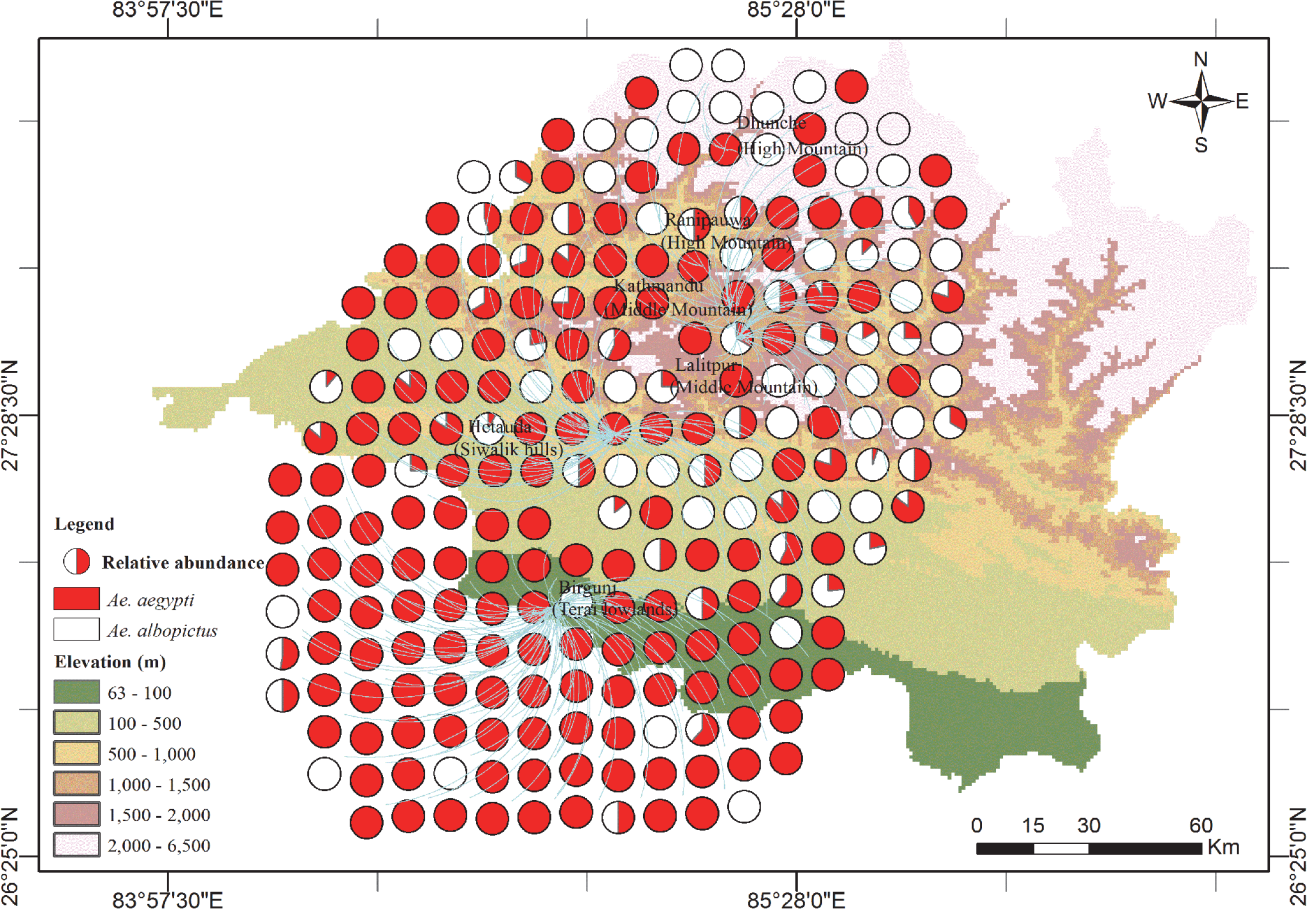
Relative abundance of *Aedes aegypti* and *Aedes albopictus* in central Nepal. Each pie-chart represents positive containers for the chikungunya and dengue virus vectors *Aedes aegypti* and *Aedes albopictus* and their co-occurrences.

Table 5 shows significant risk factors for the co-occurrence of both *Ae*. *aegypti* and *Ae*. *albopictus* in multivariate logistic regression analysis. The species were more likely to co-occur in the Siwalik and Middle Mountain regions, and were less likely to co-occur in January and February. Again, both species were more likely to co-occur outdoors. We found that *Ae*. *aegypti* dominated in the warm climate of the Terai lowlands and that the co-occurrence both species was quite high towards higher altitudes as shown in Fig. 2.

**Table 5:** Multivariate logistic regression analysis for the co-occurrence of larvae and pupae of *Aedes aegypti* and *Aedes albopictus*.

Predictor variables	Wet containers	Positive (%)	OR (95% CI)	aOR (95% CI)	*P*
**Physiographic region**					
Terai	493	1.4	1.00	1.00	
Siwalik	488	7.4	5.45 (2.37–12.54)	7.14 (3.03–16.82)	< 0.001
Middle Mountain	560	3.0	2.30 (0.93–5.67)	2.56 (1.03–6.37)	0.044
High Mountain	504	0.0	ND	ND	ND
**Month**					
Sep-11	554	2.5	1.00	1.00	
Oct-11	357	2.8	0.89 (0.39–2.06)	0.8 (0.32–1.99)	0.633
Nov-11	290	5.2	1.41 (0.66–3.02)	0.81 (0.36–1.82)	0.602
Dec-11	186	6.5	1.55 (0.69–3.47)	0.68 (0.29–1.62)	0.387
Jan-12	306	1.6	0.47 (0.17–1.34)	0.24 (0.08–0.71)	0.01
Feb-12	352	1.1	0.32 (0.1–1)	0.22 (0.07–0.71)	0.012
**Station**					
Indoor	763	2.5	1.00	1.00	
Outdoor	1282	3.2	1.96 (1.12–3.43)	3.01 (1.64–5.51)	< 0.001

OR: Odds ratio

aOR: Adjusted odds ratio

### Effect of climatic factors on the abundance of larvae of chikungunya and dengue vectors

**Fig 3:**
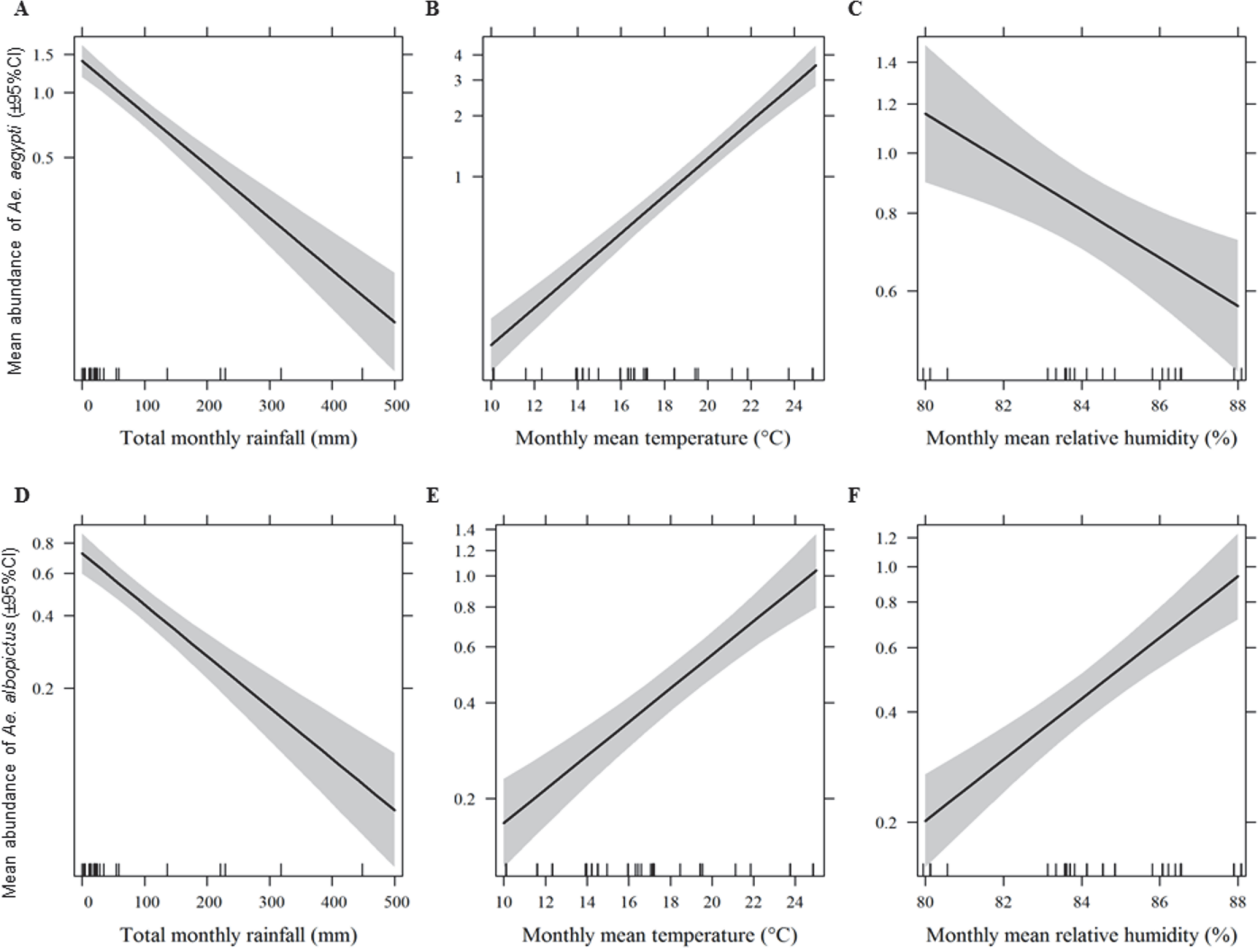
Effects of climate variables on the abundance of larvae of vector mosquitoes. Panels A, B and C show the effects of monthly total rainfall (mm), mean temperature (°C) and relative humidity (%), respectively, on the abundance of *Aedes aegypti* per container. Panels D, E and F show the effects of monthly total rainfall (mm), mean temperature (°C) and relative humidity (%), respectively, on the abundance of *Aedes albopictus* per container. Mosquito abundances are displayed in log scale.

We found significant effects of climatic variables on the mean abundance of each mosquito species (Fig. 3) and graphical presentation of observed relationship between climatic factors and abundance of *Aedes* vector in Supplementary Figure (S1 Fig.). Each degree rise in mean temperature increased *Ae*. *aegypti* abundance (β = 1.23; 95% CI = 1.18–1.29; *P*< 0.001); increased rainfall reduced abundance (β = 0.99; 95%CI = 0.99–0.99; *P*<0.001) and increased relative humidity also reduced the vector abundance (β = 0.91; 95% CI = 0.85–0.98; *P*<0.05). Likewise, an increase of the mean temperature had a positive effect (β = 1.12; 95% CI = 1.06–1.20; *P*<0.05), total rainfall had a significant negative effect (β = 0.99; 95% CI = 0.99–0.99, *P*<0.001), and relative humidity had a significant positive effect (β = 1.21; 95% CI = 1.08–1.35, *P*<0.001) on the abundance of *Ae*. *albopictus*.

## Discussion

We determined the presence and abundance of the CHIKV and DENV vectors *Ae*. *aegypti* and *Ae*. *albopictus* along an altitudinal transect from Birgunj (∼85 m asl) to Dhunche (∼2,100 m asl) in central Nepal. During the collection periods, *Ae*. *aegypti* and *Ae*. *albopictus* were commonly found up to 1,350 m asl in the Kathmandu valley and rarely found from 1,750 to 2,010 m asl in Ranipauwa and Dhunche, respectively. We also recorded *Cx*. *quinquefasciatus* as the second most abundant species throughout this study transect. This species is the principal vector of lymphatic filariasis in Nepal. The results of our physiographical, seasonal and habitat sampling allowed us to identify significant predictors of the presence of these CHIKV and DENV vectors and their co-occurrence. The abundance of these mosquito species was significantly affected by the climatic variables temperature, rainfall and relative humidity.

No data were previously available on the distribution of CHIKV and DENV vectors in the High Mountain region of Nepal (Central Himalayas). Therefore, to the best of our knowledge, this first report on this topic suggests that these vectors have already expanded their geographical range, posing health risks to mountain people in Nepal. Previously, *Ae*. *aegypti* and *Ae*. *albopictus* larvae and adults had been recorded up to the Middle Mountain region in Kathmandu Valley (∼1,350 m asl) [9,10,13,25] and their eggs (but not confirmed) up to the High Mountain region in central Nepal [25]. Our present study confirmed that those eggs were likely to have been either *Ae*. *aegypti* or *Ae*. *albopictus* and that adults of these species could not be trapped because of their low density [25]. Similar to our findings, adults of *Ae*. *aegypti*, *Ae*. *ablbopictus* and *Cx*. *quinquefasciatus* were found to be distributed up to 2000 m in eastern Nepal [39], immature *Ae*. *aegypti* were found to be distributed up to 2,130 m asl in Darjeeling, Eastern Himalayas [40], and in the city of Puebla, Mexico [41], and *Ae*. *albopictus* up to 1,300 m in the Western Himalayas [42]. In contrast, *Ae*. *aegypti* had so far only been reported below 800 m in the Western Himalayas [42,43].

The *Stegomyia* indices were able to detect the spatial variation of both species and were useful for determining general distribution patterns, seasonal changes and principal larval habitats. These indices are the chief surveillance tools of many vector control programs in CHIK and DF endemic countries worldwide [44] although their use is increasingly reported as being inadequate to measure either the risk of transmission or the effectiveness of control measures [45]. The *Stegomiya* indices of the two vectors *Ae*. *aegypti* and *Ae*. *albopictus* differed at the lower elevation sites (i.e., < 100 m, warmest region) because for *Ae*. *aegypti* oviposition, temperature suitability peaks in the warmest tropical climate whereas for *Ae*. *albopictus* the tropical climate is predicted to be less suitable due to the vector’s reduced survival at higher temperatures relative to *Ae. aegypti [46]*. Dengue fever cases had been reported previously from localities where each *Stegomyia* index was >9% in our study. Similarly, locally transmitted CHIK cases were recorded after our study in Kathmandu and in neighboring Dhading district in 2013 (>1,000 m asl) where *Ae*. *aegypti* and *Ae*. *albopictus* had been recorded previously [25] and also in the present study. Consistent to our findings, the monthly number of DF cases was also found to be correlated with *Stegomyia* indices in central Highland Province, Vietnam [47]. In contrast, DENV transmission in Singapore occurred even when the HI was less than 3% [48]. The high values of *Stegomyia* indices in central Nepal and corresponding low level of good knowledge and practice about DF prevention indicate a potential for local DENV transmission and future outbreaks as predicted in previous studies [9,12,14,15,49]. However, a recent systematic review concluded that there was little evidence of a quantifiable association between vector indices and DENV transmission to predict dengue outbreaks [44] and suggested that emphasis should be given to spatial heterogeneity and vector ecology during sampling efforts.

Our results differ from findings from a multi-site study conducted in Southeast Asian countries, where *Ae*. *albopictus* was not recorded except in small numbers in Sri Lanka [50] but are consistent with findings from the Central African Republic, India, Laos, Philippines, and Vietnam which all reported on the coexistence of *Ae*. *aegypti* and *Ae*. *albopictus* [51–56] which increases the risk of the emergence of DF epidemics [54,57]. Whereas *Ae*. *albopictus* has been known to occur in Nepal as early as the 1950s [7,8], *Ae*. *aegypti* was very recently introduced to the country and subsequently became involved in the recent DF outbreaks in Nepal [9,10,12,14]. The latter species dominated over *Ae*. *albopictus* in the lowlands, and co-occurrence in the same larval development containers was higher in the Siwalik hills and Middle Mountains compared to the lowland Terai (Fig. 2 and Table 5). These observations are consistent with the reports of displacement of *Ae*. *albopictus* by *Ae*. *aegypti* in other countries of South Asia [58–61]. Although *Cx*. *quinquefasciatus* is regarded as a domestic species that prefers polluted water and is usually found in septic tanks and drainages [62], we recorded about 21% of all collected specimens in the clean stagnant water of domestic containers, mostly discarded tires. The present findings are similar to others with respect to the artificial larval development containers of *Aedes* and *Culex* species [53,55,56,63,64] in that the wet containers that were most commonly found infested were discarded tires. Interestingly, the highest infestation rates of *Ae*. *aegypti* and *Ae*. *albopictus* separately, and of both species together, were recorded in mud pots (>18% among 8 wet containers recorded; Table 2) which had also been reported as one of the potential larval development containers of *Aedes* species in India [54]. We did not find any larvae or pupae of these *Aedes* species in natural containers. This is similar to data from Thailand where significantly higher chances of finding *Aedes* larvae were reported for artificial compared to natural containers [65]. The occurrence of *Ae*. *aegypti* or *Ae*. *albopictus*, or both species together, was significantly higher in outdoor compared to indoor containers which is consistent with results from India and Peru [54,66]. Table 2 shows certain locations within the study area were more likely to be infested indicating clustering of high risk areas (clumped distribution).

The abundance of *Ae*. *aegypti* and *Ae*. *albopictus* and, accordingly, CHIK and DF follow seasonal patterns. Our comparison of the results of the present study with those of previous ones [54,65,67,68] revealed the seasonal variation of the presence of CHIKV and DENV vectors in the study area and their significantly higher co-occurrence in the post-monsoon (September-November) compared to winter (December-February) months (Table 3–5). We also found a significant association between the presence of *Ae*. *aegypti* or *Ae*. *albopictus* and their co-occurrence with physiographic regions (i.e., the High Himalayan Region, High Mountain, Middle Mountain, Siwaliks and Terai as shown in Fig. 1) which is consistent with findings from Vietnam [53]. In univariate analysis, the presence of *Ae*. *aegypti* or *Ae*. *albopictus* and their co-occurrence were significantly associated with the type of container (discarded tire or mud pot) but did not increase the fit of the models for both species and their co-occurrence. Thus, this predictor was subsequently dropped in all models. In contrast to our findings, container type was reported to be a significant predictor in multivariate regression analysis in the Central African Republic and Laos [55,56] suggesting that other risk factors such as physiographic region, season of collection and collection site outweigh the role of container type in Nepal.

The potential effects of climatic factors, and climate change in particular, on DENV vectors and transmission have generated much debate [41,69–73]. Studies of the associations between climatic variables and DENV vectors are complex and challenging due to the dependence of the vector on humans, especially its preference for artificial containers for larval development, the availability of which in turn is influenced by local socio-economic conditions, human habitats and behaviors such as water storage practices [69,74–78]. These human related factors which act as confounders should be integrated in models to predict the future impacts of climate change on DENV vectors and transmission [41,69,79,80]. The effects of climate and other environmental changes are location-specific and it has been projected that the impact of climate change can alter the geographic distribution of disease vectors and vector-borne diseases [81–83].

We found significant associations as well as effects of temperature, relative humidity and rainfall on the abundance of *Ae*. *aegypti* and *Ae*. *albopictus* (Fig. 3). Important effects of temperature on these species and the diseases they can transmit are those that shorten the extrinsic incubation period of pathogens, lead to increases in biting frequency and extensions of the average life span of mosquitoes [3,84,85]. We found a positive linear relationship between the abundance of CHIKV and DENV vectors with mean temperature ranging from 10–25°C which is consistent with the findings of a recent meta-analysis [86] and a study on the adult vectors in Nepal [25]. We found an opposing effect of relative humidity on the abundance of *Ae*. *aegypti* and *Ae*. *albopictus* in the range between 80–90% relative humidity, with an optimum range of 83–87%. This can be partially explained due to the fact that increased relative humidity increased the abundance of immature *Ae*. *albopictus* presumably because the hatching of *Ae*. *albopictus* eggs is higher at higher relative humidity [87,88]. Increased temperature and relative humidity have been reported as being associated with an increased risk of DF in central Highland Province, Vietnam [47]. The potential impact of changing rainfall patterns is more difficult to predict due to the fact that larvae of these *Aedes* species develop in a wide range of water holding containers many of which are kept filled primarily by human action rather than natural precipitation [74,89]. In some cases, increased rainfall may increase the vector population size by creating new or better larval habitats [13] while excessive rain may eliminate habitats through flooding, thus decreasing the vector population [90,91].

In our study, decrease in rainfall was associated with increased mosquito abundance which may be related to an increasing availability of man-made larval developmental sites created by household water storage practices of people in the dry season in Nepal as reported previously [25]. In Nepal, rainfall was found to be an important factor regulating the abundance of outdoor breeding mosquito populations. A decline in the abundance of mosquitoes was observed during the monsoon, the main rainy season from June to September that may occasionally commence as early as April. Variation in distribution and abundance during the study period could be the result of climatic factors fluctuating from season to season. Seasonal changes in weather conditions can modify the blood-feeding success of mosquitoes and cause them to change their oviposition behavior depending on the availability of larval developmental habitats. Changes in microclimate due to the severity and duration of rain might therefore have positive or negative impacts on the number of mosquito eggs or larvae and pupae. On the one hand, the onset of rainfall can create additional larval developmental sites and support the growth of vegetation cover providing cool shaded microhabitats for the development of the aquatic stages and for resting adults. On the other hand, it can limit the dispersal and reproduction of these mosquitoes when it is coupled with strong winds that disturb the flight activity, leading to difficulties in finding mates, hosts and favorable larval development habitats.

The low abundance of mosquitoes in September might not only be attributed to the flushing of drains and the flooding of other outdoor breeding foci reported elsewhere [90,91], but also to increased mortality due to physical impact by heavy rain [25]. During the post-monsoon season lasting from October to November with very low or no rainfall, we observed higher numbers of *Ae*. *aegypti* and *Ae*. *albopictus* compared to September, a month with high rainfall. On the other hand, the relatively larger number of vectors collected at lower elevations may be due to increased human disruption in those areas such as rapid unplanned urbanization, many construction activities which create more artificial water holding containers such as discarded tires, cement tanks, etc. There are a number of possible explanations for the increased density at lower altitudes including the availability of the preferred hosts, favorable larval development places, and higher survival rates of immature stages leading to an increase in the number of emerging adults.

We were interested in the potential impact of climate change, especially of the more pronounced rise in temperature in the higher altitudes of Nepal, on the distribution of CHIKV and DENV vectors and transmission. The annual average temperature trend along our transect in the mountain region has been in the range of +0.2–0.7°C increase per decade since 1980 [92] and is projected to continue rising at a similar or faster rate throughout this century [93]. The annual maximum, minimum and mean temperatures of the Middle Mountain region (Khumaltar meteorological station, 1,350 m asl) are 24.4°C, 11.6°C and 18.0°C, respectively, and those of the High Mountain region (Kakani meteorological station, 2,064 m asl), 19.8°C, 10.8°C and 15.3°C [92]. More importantly, a recent study shows that the diurnal temperature range (DTR) rather than the mean temperature plays an important role in DENV transmission by *Ae*. *aegypti* and that an increasing trend of the global dengue epidemic potential is predicted in temperate regions [94]. The range of diurnal temperature in lower elevation is favorable for the mosquito breeding, hence in the present study the abundance was less as collection were farther done from the lower elevation to the higher. As temperature especially minimum temperature rise, it increases elevation ceiling as well as seasonal distribution and abundance of mosquito vectors.

We have found these two *Aedes* species (*Ae*. *aegypti* and *Ae*. *albopictus*) where the mean temperature was between 10–25°C. According to the Intergovernmental Panel on Climate Change (IPCC), the minimum temperature required for dengue virus transmission is 11.9°C and the minimum temperature for biological activity of *Aedes* mosquitoes is 6–10°C [95]. It is predicted that at mean temperatures <18°C, DENV transmission increases as DTR increases, whereas at mean temperatures >18°C, larger DTR reduces DENV transmission [96]. As climate change increases minimum temperatures, areas previously unsuitable for DENV and CHIKV transmission (< 12°C minimum temperature) may become suitable at least during the summer season in temperate regions (> 12°C minimum temperature) as has been reported elsewhere for many years [97,98].

We conclude that chikungunya and dengue virus vectors have already established populations as much as 2,000 m above sea level in Nepal and that this may be attributed to the increasing movement of people and means of transportation, import of goods from the lowlands, and environmental and climate change that has been observed over the decades in Nepal. The pronounced warming, increasing movement of people between endemic and non-endemic areas and rapid range expansion of these disease vectors in the High Mountain region, previously considered non-endemic for dengue and chikungunya fever, calls for urgent actions such as improving the capacity for differential diagnosis of febrile illness and extending and scaling-up vector-borne disease surveillance and control programs in the mountain region. The main limitation of our study is that we were not able to extend our surveys to one or several years to better understand aspects like season-to-season variation, vector dynamics and restrictions in altitudinal distribution due to niche availability. Moreover, we were unable to test for the presence of CHIKV and DENV in mosquitoes and could not link our entomological and climatic data to DF cases during the study period because no sufficiently disaggregated disease data was available. Despite these limitations, and because no such medical entomology data was available previously in Nepal, our study provides baseline information on the altitudinal distribution of CHIKV and DENV vectors and associated risk factors that is important for planning successful control operations. In addition, better knowledge of the occurrence of these vector mosquito species in the study areas may be valuable for targeted early warnings about the potential spread of the diseases, increasing the awareness of the general public and tourists, and promoting community-level as well as individual preventive and control measures.

## Supporting Information

S1 Fig Climatic factors and observed abundance of larvae of vector mosquitoes. Panels A, B and C show the relationship of monthly total rainfall (mm), mean temperature (°C) and relative humidity (%), respectively, and the abundance of *Aedes aegypti* per container. Panels D, E and F show the relationship of monthly total rainfall (mm), mean temperature (°C) and relative humidity (%), respectively, and the abundance of *Aedes albopictus* per container.
(TIF)

S1 Table Abundance of *Culex quinquefasciatus* in central Nepal. This table shows abundance data recorded in fresh water holding containers only and do not cover data from the potential larval developmental habitats such as polluted water sources, septic tanks, drainage etc. The “No. identified to species” refers the total number of larvae from all the wet containers at that location that were identified to species. The last column indicates the percentage of containers that contained *Cx*. *quinquefasciatus* and the 95% confidence interval (CI).
(DOCX)
