# Supplementary figures and images for: Risk Factors for the Presence of Chikungunya and Dengue Vectors (Aedes aegypti and Aedes albopictus), Their Altitudinal Distribution and Climatic Determinants of Their Abundance in Central Nepal
Source: PLoS Negl Trop Dis. 2015 Mar 16;9(3):e0003545. doi: 10.1371/journal.pntd.0003545 (PMC4361564; doi:10.1371/journal.pntd.0003545)

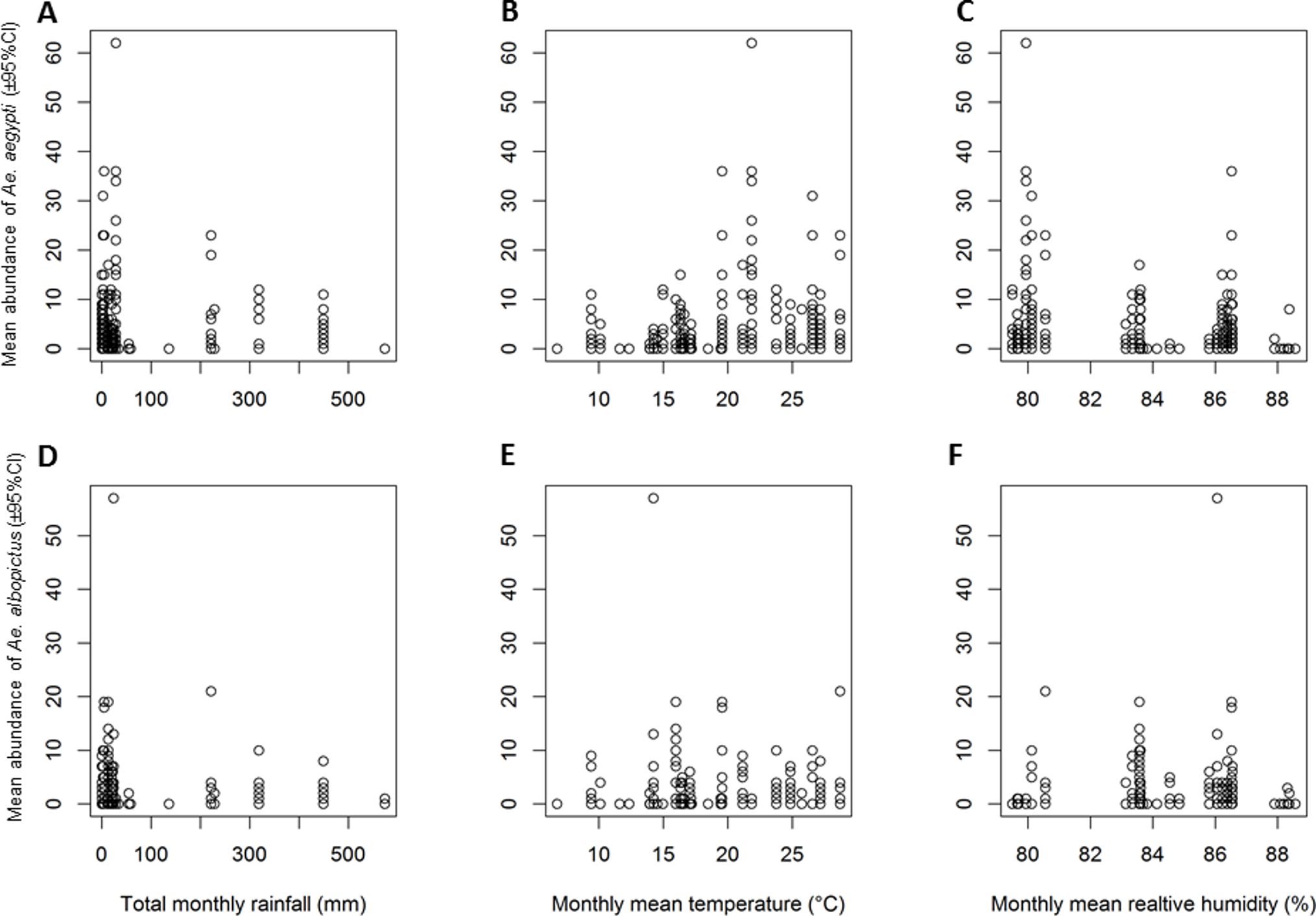

Supplement: S1 Fig — Panels A, B and C show the relationship of monthly total rainfall (mm), mean temperature (°C) and relative humidity (%), respectively, and the abundance of Aedes aegypti per container. Panels D, E and F show the relationship of monthly total rainfall (mm), mean temperature (°C) and relative humidity (%), respectively, and the abundance of Aedes albopictus per container. (TIF) [file pntd.0003545.s001.tif]
