# Supplementary material for: Risk Factors for the Presence of Chikungunya and Dengue Vectors (Aedes aegypti and Aedes albopictus), Their Altitudinal Distribution and Climatic Determinants of Their Abundance in Central Nepal
Source: PLoS Negl Trop Dis. 2015 Mar 16;9(3):e0003545. doi: 10.1371/journal.pntd.0003545 (PMC4361564; doi:10.1371/journal.pntd.0003545)
Supplement: S1 Table — This table shows abundance data recorded in fresh water holding containers only and do not cover data from the potential larval developmental habitats such as polluted water sources, septic tanks, drainage etc. The “No. identified to species” refers the total number of larvae from all the wet containers at that location that were identified to species. The last column indicates the percentage of containers that contained Cx. quinquefasciatus and the 95% confidence interval (CI). (DOCX) [file pntd.0003545.s002.docx]

| S1Table. Abundance of *Culex quinqufasciatus* in central Nepal | | | |  |
| --- | --- | --- | --- | --- |
| Localities/Study district | Physiographic region | Wet containers inspected | No. identified to species | Proportion of positive containers (95% CI) |
| Birgunj (90m) | Terai | 493 | 193 | 9.9 (7.5 - 13.0) |
| Hetauda (460m) | Siwalik | 488 | 85 | 4.3 (2.7 - 6.6) |
| Kathmandu (1310m) | Middle Mountain | 307 | 21 | 3.6 (1.9 - 6.5) |
| Lalitpur (1320m) | Middle Mountain | 253 | 191 | 13.8 ( 9.4 - 18.9) |
| Ranipauwa (1850m) | High Mountain | 238 | 62 | 6.7 (4.0 - 10.9) |
| Dhunche (2100m) | High Mountain | 266 | 134 | 9.0 (6.0 - 13.2) |
| Grand Total |  | 2045 | 686 | 7.6 (6.5 - 8.9) |
